# Estimation of Cognitive Performance Based on Premorbid Intelligence in Parkinson’s Disease

**DOI:** 10.3233/JPD-202142

**Published:** 2020-10-27

**Authors:** Rocio Del Pino, Maria Díez-Cirarda, Javier Peña, Naroa Ibarretxe-Bilbao, Natalia Ojeda

**Affiliations:** a Neurodegenerative Diseases Group, Biocruces Bizkaia Health Research Institute, Barakaldo, Spain; b Research Imaging Centre, Campbell Family Mental Health Research Institute, Centre for Addiction and Mental Health, University of Toronto, Ontario, Canada; c Department of Methods and Experimental Psychology, University of Deusto, Bilbao, Spain

**Keywords:** Neuropsychological assessment, Parkinson’s disease, premorbid intelligence, pseudo-words, WAT

## Abstract

**Background:**

The estimation of premorbid intelligence (PI) is needed for an accurate diagnosis.

**Objective:**

This study aimed to estimate the cognitive performance taking into account the PI in Parkinson’s disease (PD) compared to healthy controls (HC); and to analyze the discrepancies between the current and the predicted cognitive performance based on the PI.

**Method:**

Semantic fluency, verbal and visual memory, and executive functions were assessed in 39 PD and 162 HC. A linear regression model was used to analyze the discrepancies between the predicted cognitive performance and the current raw scores through PI variables (Word Accentuation Test (WAT), Pseudo-Words (PW) Reading subtest from PROLEC-R, age, and years of education). ROC analyses were performed to assess their diagnostic properties.

**Results:**

Significant differences were found in the raw cognitive scores between patients and HC [semantic fluency (*t* = 6.07; *p* < 0.001), verbal memory (*t* = 6.63; *p* < 0.001), and executive functions (*t* = 2.57; *p* = 0.013), and in visual memory (*t* = 1.97; *p* = 0.055 marginally significant)]. Compared to HC, PD patients presented higher discrepancies between the predicted cognitive performance and the raw scores in semantic fluency, verbal memory, visual memory, executive functions (AUC = 0.78, 0.78; 0.64, 0.61, respectively).

**Conclusion:**

The magnitude of the discrepancies scores between the current and the predicted cognitive performance based on PI indicates the presence of cognitive decline in the specific cognitive domain in PD patients. This study underlines the usefulness of premorbid measures and variables, such as WAT, PW, age, and years of education, to more accurately estimate the cognitive performance in PD.

## INTRODUCTION

Neuropsychologists often need to compare a person’s actual performance with his/her expected performance in order to infer whether cognitive impairment has been developed [1–3]. However, unless the person was assessed before the onset of an illness or injury, his or her premorbid abilities have to be estimated [1, 2, 4]. The estimation of premorbid intelligence (PI) is needed for researchers and clinicians who work with different pathologies (e.g., schizophrenia or dementia) [5–7] or healthy people who suffer cognitive decline (e.g., aging) [4, 8] because of its relevance in an acute early diagnosis and rehabilitation process. According to Barnett et al. (2006), high PI is could be a protective factor against the development or expression of neurodegenerative or neuropsychiatric diseases.

There are several methods to assess PI. The most common method is measuring the person’s reading ability because highly correlated with almost all other cognitive tasks, it is relatively unaffected by brain dysfunction [2, 9], and it plays a central role in many cognitive abilities [10]. The most common instrument to assess reading ability in English speaking populations is the *National Adult Reading Test* (NART) [11], later revised for North American people (NART-R) [12]. The Spanish adaptation of this test is the Word Accentuation Test (WAT) [6, 13]. This version assesses the ability to correctly read infrequent words written without accent marks. Another distinctive component of the reading skills can be assessed by the use of novel letter strings [nonwords, non-existing words or pseudo-words (PW)], whose pronunciation depends on the reader’s general knowledge of correspondences between spelling patterns and pronunciations [14]. This method to assess PI is effective regardless presence of cognitive impairment or even dementia, since it is well established that when the dementia progresses, semantic memory deteriorates but not the phonological knowledge [14, 15].

Another common method to assess PI is based on the individual’s demographic characteristics including variables such as age or sex into multiple regression models [2, 6, 16, 17]. Variables such as years of education or occupation could also significantly affect premorbid abilities before the onset of the disease. Therefore, both demographic and educational variables should be taken into account to accurately evaluate the PI of the person assessed [18]. Combining both sociodemographic variables and performance on word reading tests yields a more accurate assessment of PI than either one approach alone [16, 18, 19]. However, even if these methodologies have been studied in different pathologies such as dementia [6, 20] or schizophrenia [7] very few studies have addressed this subject in PD [21]. To our knowledge, this study is the first study to evaluate cognitive decline in PD considering the individual’s PI level.

PD patients have shown cognitive impairment in a wide range of cognitive domains such as executive functions, visuospatial ability, memory and semantic fluency [22]. Henry and Crawford (2004) conducted a meta-analysis finding that PD patients showed more impairment in semantic fluency than phonemic fluency and suggested that PD appears to be associated with verbal memory deficits [23]. These cognitive deficits are present from the early stages of the disease [3], thus, the early detection of these deficits is suggested to help in the early PD diagnosis [24]. In this process, a reliable neuropsychological diagnosis, sensitive to individual characteristics of each person, is a key factor that involves a correct neuropsychological assessment but also an accurate assessment of patients’ PI in order to carry out a correct diagnosis and not misclassify patients [25]. However, the PD literature on the influence of PI assessment in the correct cognitive diagnosis is scarce [21, 26].

Therefore, this study first aimed to estimate the cognitive performance (i.e., predicted cognitive performance) in PD patients compared to HC taking into account PI variables (WAT, the PW reading subtest, age, and education). The second objective was to analyze the discrepancies between the current (raw scores) and the predicted cognitive performance based on the PI in both PD and HC groups; The third aim was to analyze the sensitivity and specificity of the obtained discrepancy scores.

## METHODS

### Participants

Thirty-nine PD patients were recruited from the Department of Neurology at the Hospital of Galdakao and from the PD Biscay Association (ASPARBI). We also included 162 healthy controls (HC). HC sample was selected from the database of the Normacog study [27].

PD patients were enrolled in the study if they fulfilled the UK PD Society Brain Bank diagnostic criteria. Other inclusion criteria were: i) age between 45–75; ii) Hoehn and Yahr disease stage <3 [28]. The exclusion criteria for both groups were as follow: i) the presence of dementia as defined by the DSM-V [29]; ii) sensory limitations (visual or auditory) which cannot be satisfactorily compensated by corrections (glasses or hearing aids); iii) the presence of other neurological illness/injury (traumatic brain injury); iv) unstable psychiatric disorders (e.g., schizophrenia); v) presence of depression evaluated with the Geriatric Depression Scale (scores > 5) [30]. Their Levodopa equivalent daily dose was registered [31]. Clinical assessment for PD patients was done by a neurologist and included Hoehn & Yahr scale and Unified PD Rating Scale (UPDRS) [32]. The clinical and sociodemographic characteristics of the sample are shown in Table 1.

**Table 1:** Sociodemographic and clinical characteristics of the HC and PD sample

	HC (*n* = 162) M (SD)	PD (*n* = 39) M (SD)	Statistic	*p*	95% CI		Cohen’s *d*
					LL	UL
Age	66.81 (7.26)	68.00 (6.35)	*t* = –1.021	0.311	–3.52	1.13	0.18
Years of education	9.83 (5.56)	10.44 (4.81)	*t* = –0.622	0.535	–2.51	1.30	0.11
Sex (Male)	75 (46.3%)	24 (61.5%)	*χ*^2^ = 2.922	0.109
Geriatric Depression Scale	2.21 (2.19)	2.13 (2.66)	*t* = –0.200	0.842	–0.72	0.88	0.03
IADL	7.67 (0.77)	6.97 (1.26)	*t* = 3.288	0.002	0.27	1.12	0.58
UPDRS III	–	21.79 (11.00)	–	–
LEDD	–	788.85 (435.30)	–	–
Disease Evolution (y)	–	6.53 (4.99)	–	–
Hoehn &Yahr	–	1.84 (0.43)	–	–
1	–	6	–	–
1.5	–	3	–	–
2	–	28	–	–
2.5	–	1	–	–
3	–	1	–	–

*Note.* Values are expressed as mean (S.D) unless otherwise noted. PD, Parkinson’s disease; HC, Healthy controls; CI, Confidence Interval; LL, Lower limit; UL, Upper Limit; IADL, Instrumental Activities of Daily Living; UPDRS, Unified Parkinson Disease Rating Scale; LEDD, Levodopa Equivalent Daily dose.

### Neuropsychological measures

The WAT [6, 13] is the Spanish adaptation of the NART-R [12]. The NART-R consists on reading aloud 61 irregular words and relies on the assumption that correct pronunciation of irregular words depends on previous encounters with the word [33]. However, the Spanish language is considered a “transparent” language because the correspondence between graphemes and phonemes is very consistent. Nevertheless, the lexical stress assignment is a source of irregularity when people read aloud in Spanish [34]. The most frequent stress pattern in multi-syllabic words is on the penultimate syllable, although there are other regularities such as words ending in consonants other than /n/ or /s/. Words that do not follow these regularities have an orthographic stress mark that indicates the syllable that should be accentuated. Hence, if the stress mark is not written, the correct pronunciation of these words requires previous knowledge of the word. Therefore, the WAT assesses the PI of Spanish speakers by correctly reading aloud 30 low frequency words whose graphic accents have been removed [6, 13].

The pseudo-words (PW) reading test is a subtest from PROLEC-R (*Baterí a de evaluación de procesos lectores-revisada*; Battery for Reading Processes Assessment-Revised) [35] that has been adapted for Spanish healthy adults [18, 27]. It aims to assess reading ability and fluency through the sublexical pathway by reading each pseudoword (or non-existent words) aloud correctly [35]. The Spanish language has the peculiarity that written words (known or not known), even PW, can be read aloud by the reader generating the sounds from letters, even by children who have only recently begun reading [34].

All participants, HC and patients with PD, underwent a neuropsychological battery including the following cognitive domains: semantic fluency (Animals + Supermarket) [36]; verbal memory (Hopkins Verbal Learning Test-Revised: HVLT-R Recall) [37]; visual memory (Brief Visuospatial Memory Test-Revised: BVMT-R Recall) [38]; naming (the abbreviated version of the Boston Naming Test: BNT) [39]; processing speed (Salthouse Perceptual Comparison Test: SPCT total) [40]; attention (Brief Test of Attention: BTA total) [38]; and executive functions (Trail Making Test part B: TMT-B) [41]. The neuropsychological assessment of patients was done in the medication ON state.

### Ethics statement

The study protocol was approved by the Research Ethics Committee at the University of Deusto and the Research Ethics Committee at the Basque Health System in Spain. All subjects were volunteers and provided written informed consent prior to their participation in the study, in accordance with the Declaration of Helsinki.

### Statistical analyses

Data were analysed using the Statistical Package for the Social Science (SPSS), version 20. Kolmogorov-Smirnov was used to test normal distribution of variables. Demographic, clinical and cognitive variables were analysed with t test and chi-squared test for categorical variables. Pearson’s correlation was used for correlation analyses between PI and cognitive variables. TMT-B scores were recoded so that higher scores indicated better cognitive performance.

To estimate the cognitive performance using PI variables (i.e., predicted cognitive performance: WAT, PW, age and years of education), the following linear regression equation was obtained in the HC group [6]:

$$\begin{array}{cc} & \mathrm{Congnitive}\;\mathrm{performanc}e_{\mathrm{predicted}}=\\ & \;\left(B*\mathrm{WAT}\right)+\left(B*\mathrm{PW}\right)\\ & \;+\left(B*\mathrm{age}\right)+\left(B*\mathrm{education}\right)+K \end{array}$$

The discrepancies between the predicted scores and the raw scores were calculated for each cognitive domain, subtracting the raw score from the predicted score [6]. The magnitude of these discrepancies reveals the degree of cognitive impairment (higher discrepancy indicates greater cognitive impairment) and could be applied to analyze the level of cognitive decline in PD according to the performance of the HC group. The mean discrepancies of PD and HC were compared. Lastly, the receiver operating characteristic (ROC) curves for the discrepancies were calculated. The areas under the curve were compared. The optimum cut-off scores were established based on the Youden Index [42], and the sensitivity (Se) and specificity (Sp) were determined.

## RESULTS

The sociodemographic and clinical characteristics of the PD and HC are shown in Table 1. No significant differences were found in sociodemographic characteristics such as age or years of education between groups.

**Fig. 1:**
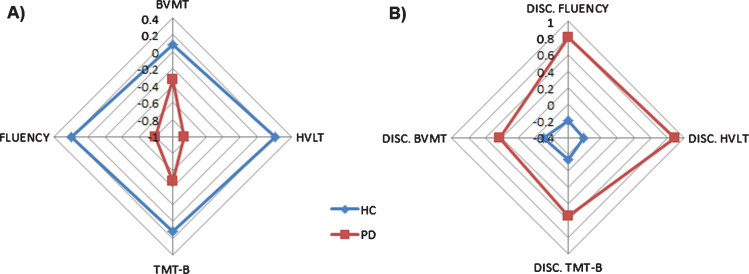
Differences in cognitive performance in both raw scores (A) and discrepancy scores (B) are shown for PD and HC groups. A) Raw score differences between PD and HC. Scores are shown in *z*-scores. B) Discrepancy scores differences between PD and HC. Scores are shown in *z*-scores.

Regarding the neuropsychological assessment, there were significant differences between the PD and HC groups in the following cognitive domains: semantic fluency, verbal memory and executive functions. In addition, marginal significant differences were found in visual memory between groups (see Table 2, Fig. 1).

**Table 2:** Neuropsychological characteristics of the HC and PD sample

	HC (*n* = 162) M (SD)	PD (*n* = 39) M (SD)	T	*P*	95% CI		Cohen’s *d*
					LL	UL
WAT	22.25 (6.56)	20.46 (7.56)	1.48	0.141	–0.595	4.16	0.26
PW	34.83 (5.71)	34.77 (6.25)	0.05	0.956	–1.99	2.10	0.008
Attention (BTA)	14.00 (4.85)	13.27 (4.76)	0.83	0.409	–1.01	2.47	0.14
Processing Speed (SPCT Total)	19.19 (8.16)	18.74 (7.76)	0.31	0.759	–2.40	3.29	0.05
Naming (BNT)	11.70 (3.03)	11.67 (2.50)	0.08	0.940	–0.894	0.964	0.01
Semantic Fluency (Animals &Supermarket)	39.88 (8.45)	30.41 (9.90)	**6.07**	**<0.001**	6.39	12.55	**1.08**
Verbal Memory (HVLT_R Recall)	7.81 (2.94)	4.15 (3.65)	**6.63**	**<0.001**	2.39	4.92	**1.18**
Visual Memory (BVMT_R Recall)	6.50 (2.99)	5.18 (3.93)	**1.97**	**0.055^**MS**^**	0.02	2.67	**0.35**
Executive Functions (TMT_B)	–141.92 (65.17)	–183.90 (96.83)	**2.57**	**0.013**	–74.87	–9.08	**0.45**

*Note.* Values are expressed as mean (SD) unless otherwise noted. PD, Parkinson’s disease; HC, Healthy controls; CI, Confidence Interval; LL, Lower limit; UL, Upper Limit; WAT, Word Accentuation Test, Spanish version of NART; PW, Pseudo-Words subtest form PROLEC-R; HVLT-R, Hopkins verbal learning Test-Revised; BVMT-R, Brief Visuospatial Memory Test-Revised; BNT, Boston Naming Test; SPCT Total, Salthouse Perceptual Comparison Test Total; BTA, Brief Test of Attention; TMT_B, Trail Making Test Part B; ^MS^, Marginally significant.

Correlations between the PI variables (WAT, PW, age, and years of education) and the cognitive domains are shown in Table 3 and Supplementary Figure 1. All cognitive domains correlated significantly with the PI variables at *p* < 0.001.

**Table 3:** Relationship between cognitive domains and PI variables

Cognitive domains	WAT	PW	Age	Years of Education
	*r*	*r*	*r*	*r*
Semantic Fluency (Animals &Supermarket)	0.44	0.36	–0.42	0.34
Verbal Memory (HVLT_R Recall)	0.35	0.20	–0.33	0.24
Visual Memory (BVMT_R Recall)	0.57	0.41	–0.44	0.59
Executive Functions (TMT-B)	0.54	0.43	–0.48	0.58

r > 0.5; r > 0.4; r > 0.3; r > 0.2. *Note.* WAT, Word Accentuation Test, Spanish version of NART; PW, Pseudo-Words subtest form PROLEC-R; HVLT-R, Hopkins verbal learning Test-Revised; BVMT-R, Brief Visuospatial Memory Test-Revised; TMT_B, Trail Making Test Part B.

### Estimation of cognitive performance based on PI variables

The previous cognitive domains that showed significant differences between PD and HC were included in the analysis of the estimation of cognitive performance using the PI variables. First, for each cognitive domain, a linear regression equation based on the HC group was used to estimate the predicted cognitive performance.

$$\begin{array}{cc} & \mathrm{Semantic}\;\mathrm{Fluenc}y_{\mathrm{predicted}}=\\ & \;\;\left(0.139*\mathrm{WAT}\right)+\left(0.268*\mathrm{PW}\right)+\left(-0.356*\mathrm{age}\right)\\ & \;+\left(0.189*\mathrm{years}\;\mathrm{of}\;\mathrm{education}\right)+49.359 \end{array}$$

$$\begin{array}{cc} & \mathrm{Verbal}\;\mathrm{Memor}y_{\mathrm{predicted}}=\\ & \;\;\left(0.054*\mathrm{WAT}\right)+\left(0.002*\mathrm{PW}\right)+\left(-0.119*\mathrm{age}\right)\\ & \;+\left(0.022*\mathrm{years}\;\mathrm{of}\;\mathrm{education}\right)+14.295 \end{array}$$

$$\begin{array}{cc} & \mathrm{Visual}\;\mathrm{Memor}y_{\mathrm{predicted}}=\\ & \;\;\left(0.047*\mathrm{WAT}\right)+\left(0.048*\mathrm{PW}\right)+\left(-0.088*\mathrm{age}\right)\\ & \;+\left(0.227*\mathrm{years}\;\mathrm{of}\;\mathrm{education}\right)+7.410 \end{array}$$

$$\begin{array}{cc} & \mathrm{Executive}\;\mathrm{Function}s_{\mathrm{predicted}}=\\ & \;\;\left(0.856*\mathrm{WAT}\right)+\left(1.640*\mathrm{PW}\right)+\left(-3.058*\mathrm{age}\right)\\ & \;+\left(3.872*\mathrm{years}\;\mathrm{of}\;\mathrm{education}\right)+\left(-53.707\right) \end{array}$$

Secondly, these newly created variables were used to calculate the discrepancy between the raw score and their predicted score in each cognitive domain for both HC and PD groups.

**Fig. 2:**
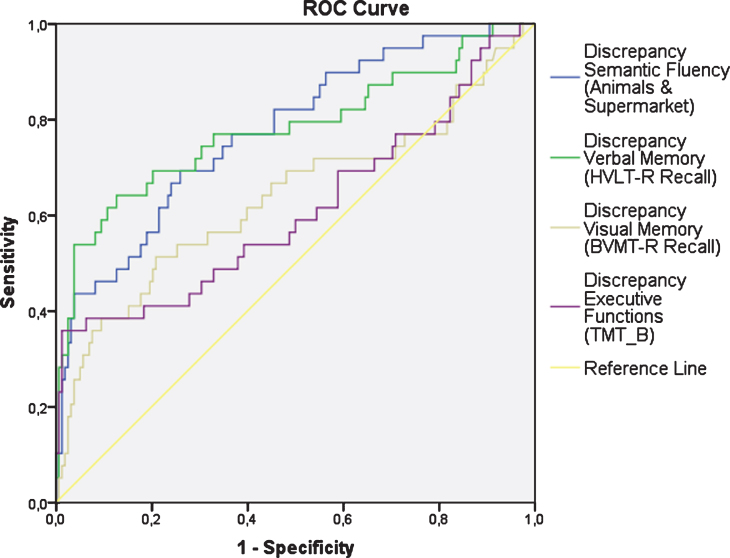
ROC curves for the discrepancies in the cognitive domains.

Compared to HC, the discrepancies between the raw cognitive scores and the predicted cognitive scores based on the PI variables were significantly higher in the PD group (Table 4 and Fig. 1). Figure 2 shows the ROC curves for these discrepancies. The area under the curve (AUC) was 0.78 (*p* < 0.001) for Semantic Fluency (Se = 0.61; Sp = 0.85), 95% Confidence interval (CI) [0.69, 0.86]; AUC = 0.78 (*p* < 0.001) for Verbal Memory (Se = 0.64; Sp = 0.87), 95% CI [0.68, 0.87]; AUC = 0.64 (*p* = 0.008) for Visual Memory (Se = 0.51; Sp = 0.79), 95% CI [0.52, 0.74]; and AUC = 0.61 (*p* = 0.03) for Executive Functions (Se = 0.36; Sp = 0.99), 95% CI [0.49, 0.72].

**Table 4:** Discrepancies between cognitive domains predicted and their raw scores

	HC (*n* = 162) M (SD)	PD (*n* = 39) M (SD)	T	*P*	95% CI		Cohen’s *d*
					LL	UL
Semantic Fluency_p_–Semantic Fluency_rs_	0.15 (7.82)	8.87 (8.56)	**–6.13**	**<0.001**	–11.52	–5.91	–1.09
Verbal Memory_p_–Verbal Memory_rs_	0.01(2.70)	3.45 (3.32)	**–5.99**	**<0.001**	–4.59	–2.28	–1.22
Visual Memory_p_–Visual Memory_rs_	–0.03 (2.23)	1.25 (2.95)	**–2.54**	**0.014**	–2.29	–0.27	–0.54
Executive Functions_p_–Executive Functions_rs_	12.98 (48.32)	52.39 (81.93)	**–2.88**	**0.006**	–66.95	–11.87	–0.70

*Note.* Values are expressed as mean (SD) unless otherwise noted; _p_, predicted; _rs_, raw scores; PD, Parkinson’s Disease; HC, Healthy Controls; PI, Premorbid Intelligence; CI, Confidence Interval; LL, Lower limit; UL, Upper Limit.

## DISCUSSION

This is the first study in PD that aimed to estimate the cognitive performance taking into account PI variables and to analyze the discrepancies between the current and the predicted cognitive performance based on the PI in both PD and HC groups.

The major finding of this study was the usefulness of two objective and very common PI measures such as the WAT and the PW reading subtest from PROLEC-R, adding 2 sociodemographic variables such as age and years of education, to obtain an accurate prediction of neuropsychological performance, specific to each individual patient, and that adds relevant information to the raw cognitive scores in PD patients.

As expected, there were significant differences in diverse cognitive domains such as semantic fluency, verbal and visual memory or executive functions in the raw scores. These cognitive domains are common features that are usually impaired in PD [22]. Specifically, PD patients usually present semantic fluency and verbal memory problems even in the early stages of the illness [23, 43]. Several authors have found that PI is related to these cognitive domains (for example, word fluency and memory) in healthy adults [44] and patients diagnosed with mild dementia [6] or moderate cognitive impairment [4]. Therefore, this study analysed the influence of PI in semantic fluency, verbal and visual memory, and executive functions. The discrepancies obtained between the predicted cognitive performance and the current cognitive performance pointed out the extent of the cognitive impairment in semantic fluency, verbal and visual memory, and executive functions in non-demented PD patients. The largest effect size and AUC was found in semantic fluency and verbal memory, while it showed to be medium to large in visual memory and executive functions. A possible explanation could be related to the fact that semantic fluency and verbal memory are tasks with verbal content and the nature of the WAT and PW is reading ability. This study suggested that these PI measures (WAT, PW, age, and years of education) could be adequate to estimate the predicted cognitive performance of PD patients and to compare it with their current performance [2, 6, 45]. Hence, the analysis of the discrepancies in different cognitive domains, taking into account the sociodemographic characteristics of the patients and their PI, will add relevant information about the cognitive decline for each specific patient, and help to detect cognitive impairment in PD [45, 46] and take correct clinical decision afterwards.

In fact, the regression equation proposed in this study showed high specificity and good sensitivity in semantic fluency and memory (verbal and visual), to correctly identify HC without cognitive impairment and PD patients who had these domains impaired. Executive functions, on the other hand, showed high specificity but medium-low sensitivity. These instruments are very brief and easy to administer and add value to the clinical decision process.

The method most widely used to assess PI is through reading ability and lexical access since reading ability is a skill that will be developed during one’s lifetime, unless brain damage appears in specific areas of language [4, 47, 48] and it is an adequate and effective method to assess premorbid functioning [4, 5, 44, 47]. In fact, the results of this study showed significant correlations between the WAT and all cognitive domains assessed, emphasizing visual memory, and executive functions. Moreover, reading PW also offers a good way to assess PI [14, 15, 49]. This could be because the sub-lexical pathway is more basic than the lexical pathway [35]. However, although each cognitive domain correlated significantly with PW, these correlations were below 0.50. Semantic fluency, visual memory, and executive functions had a moderate correlation with PW (between 0.30 and 0.49). Furthermore, demographic variables such as age and years of education have also been used to assess premorbid functioning [8, 45, 50, 51] because these data are independent of the patient’s cognitive decline [2, 8]. The results showed high correlations between years of education and visual memory, and executive functions. As has been said before, the rest of the cognitive domains assessed also correlated significantly with age and education, but these correlations were moderate (below 0.50).

Therefore, demographic data combined with both word reading test such as the WAT and the PW reading test from PROLEC-R, are good measures to assess PI in HC and PD patients. This combination was previously proposed in healthy adults, in dementia [5, 8, 44], and in patients with schizophrenia [52]. However, to our knowledge, this is the first study that demonstrated the relevance of considering the PI to estimate the predicted cognitive performance in PD.

It is also important to note that this study has several limitations. Regarding the PD sample, the sample size was small, patients were in the mild to moderate Hoehn and Yahr stages of the disease, and even if the LEDD was registered, other kind of medication that could affect cognition were not registered. Hence, future studies should recruit a larger sample of PD patients, including PD patients in more advanced stages of the disease and PD patients with dementia, and also register any medication that could have an impact on cognition. However, since the detection of potential cognitive decline is more relevant in early stages, we focused on non-demented PD patients with fewer years of disease evolution. Moreover, future studies could assess the influence of these PI variables in other cognitive domains such as working memory or visuospatial abilities. Another limitation could be the difficulty to generalize these results to other samples of Spanish speaking groups from different countries as there are so many relevant differences in the use of vocabulary in the varieties of the Spanish language used in countries other than Spain. Also, it would be interesting to carry out future studies with this methodology in different populations such as other neurodegenerative diseases as well as psychiatric disorders. High PI seems to be a protective factor against neurological conditions, whilst low PI could be a vulnerability factor that lowers the threshold for the symptoms, functional impairment and clinical presentation [53].

Findings of the present study suggested that the magnitude of the discrepancies between the current cognitive performance and the predicted cognitive performance revealed the degree of cognitive impairment in patients with PD, and add relevant information that could be used for a more accurate identification of cognitive decline in early phases of PD. Consequently, a more precise interpretation of the person’s cognitive performance and variations is proposed comparing the current cognitive performance and the predicted performance based on the suggested PI variables (WAT, PW, age and years of education). This measure would be specific to each patient and would add relevant information about the patient’s cognitive decline also longitudinally.

## CONFLICT OF INTEREST

No potential conflict of interest was reported by the authors.

## Supplementary Material

Supplementary Material
