# Supplementary material for: Estimation of Cognitive Performance Based on Premorbid Intelligence in Parkinson’s Disease
Source: J Parkinsons Dis. 2020 Oct 27;10(4):1717–25. doi: 10.3233/JPD-202142 (PMC7683044; doi:10.3233/JPD-202142)
Supplement: Supplementary Material [file jpd-10-jpd202142-s001.pdf]

Supplementary Material

Estimation of Cognitive Performance Based on Premorbid Intelligence in Parkinson's Disease

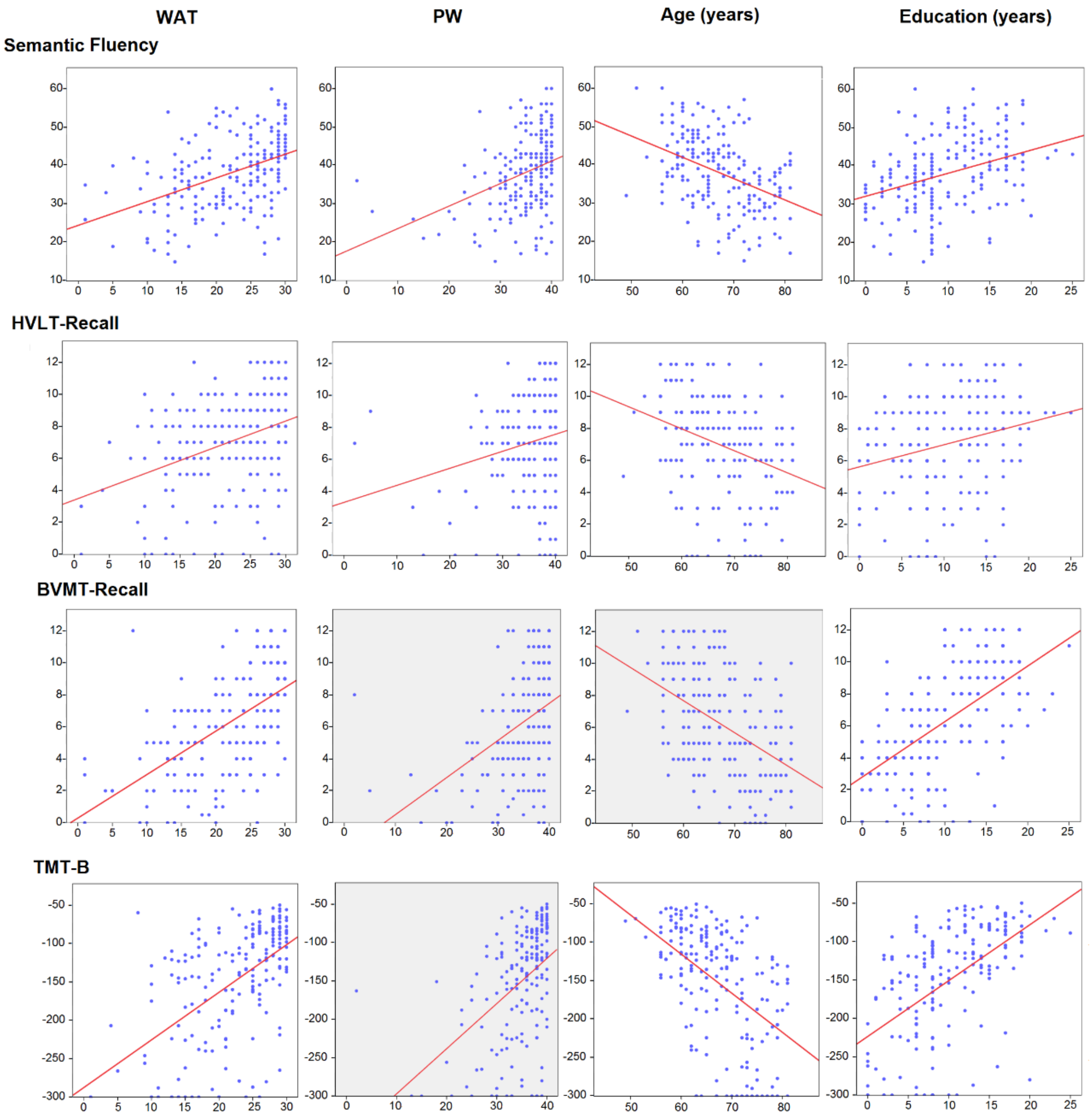

Supplementary Figure 1. Relationship between cognitive domains and PI variables
